# Chromoblastomycosis Due to a Never-before-Seen Dematiaceous Fungus in a Kidney Transplant Patient

**DOI:** 10.3390/microorganisms9102139

**Published:** 2021-10-13

**Authors:** Hélène Guegan, Marine Cailleaux, François Le Gall, Florence Robert-Gangneux, Jean-Pierre Gangneux

**Affiliations:** 1 Univ Rennes, CHU Rennes, Inserm, EHESP, Irset (Institut de Recherche en Santé, Environnement et Travail)—UMR_S 1085, F-35000 Rennes, France; florence.robert-gangneux@univ-rennes1.fr (F.R.-G.); jean-pierre.gangneux@univ-rennes1.fr (J.-P.G.); 2 Infectious Diseases and Intensive Care Unit, CHU Rennes, F-35033 Rennes, France; marine.cailleaux@chu-rennes.fr; 3 Department of Pathology, CHU Rennes, F-35033 Rennes, France; francois.le.gall@chu-rennes.fr

**Keywords:** chromoblastomycosis, Kirschsteiniotheliales, Dothideomycetes, fumagoid cell, kidney transplant

## Abstract

Chromoblastomycosis is a neglected fungal infection of the epidermis and subcutaneous tissue that predominates in tropical areas and results from the traumatic inoculation of environmental dematiaceous filamentous fungi. We describe the case of an immunosuppressed patient diagnosed with foot chromoblastomycosis due to an uncommon dematiaceous fungus. A 52-year-old Congolese kidney transplant woman presented with a painful lesion located on the foot. No trauma to the lower limbs was reported during the previous months. She lived in France and had not returned to the Congo over the previous eight years. Histology and mycological examination from skin biopsy revealed swollen dark filaments associated with dematiaceous muriform cells, pathognomonic of chromoblastomycosis. Cultures grew with dark pigmented colonies, yielding poor microscopic features. The phylogenetic analysis confirmed that the isolate was a member of Kirschsteiniotheliales (Dothideomycetes) and unrelated to the Chaetotyriales, of which most species commonly responsible for chromoblastomycosis belong. As there was no bone spreading, excision surgery of the entire lesion followed by liposomal amphotericin B therapy resulted in complete healing after six months. This original case illustrates the potential diversity of environmental dematiaceous fungi responsible for phaeohyphomycosis, especially chromoblastomycosis, and the need to send samples to mycology labs for appropriate diagnosis.

## 1. Introduction

Chromoblastomycosis is a neglected endemic fungal disease that leads to chronic cutaneous and subcutaneous mycoses. Such mycoses are highly prevalent in tropical and subtropical regions, especially Asia, Latin America, and Madagascar [1,2,3]. These infections predominantly affect active immunocompetent adult males working in agricultural fields and are generally acquired through accidental inoculation of plant- or soil-associated fungi by thorns or wound contamination [4]. The disease hallmark is the presence of single or clustered muriform cells, also called fumagoid cells, sclerotic cells or Medlar bodies embedded in a granulomatous and suppurative tissue, that serve as invasive forms in living tissue. This is the most predominant form, which triggers an intense inflammatory pattern responsible for the chronic inflammatory reaction seen in most patients [5]. Chromoblastomycosis must be distinguished from the closely related phaeohyphomycosis, which is globally distributed and more commonly responsible for rapidly evolving multiple lesions in immunosuppressed hosts, in whom typically muriform elements are not detected. We describe the case of an immunosuppressed patient diagnosed with foot chromoblastomycosis due to an uncommon dematiaceous fungus.

## 2. Case Report

A 52-year-old Congolese woman presented to our hospital with a painful lesion located on the external side of the left foot that had been evolving for two months. The unique abscess-like lesion measured 2 cm in diameter and produced a chronic sero-hematic discharge.

In her medical history, the patient had had a kidney transplant two years before and still received tacrolimus (15.5 mg/d), mycophenolate mofetil (1 g/d), and corticosteroid (5 mg/d). No trauma to the lower limbs was reported during the previous months. She lived in France and had not returned to the Congo over the previous eight years. At the time of the initial consultation, she had just returned from a trip to Morocco, where she had spent a few months.

The first skin biopsy was poorly contributory due to superficial sampling. Histopathological examination revealed the presence of a single fungal hypha with no clear distinction between yeast or filamentous fungus.

**Figure 1:**
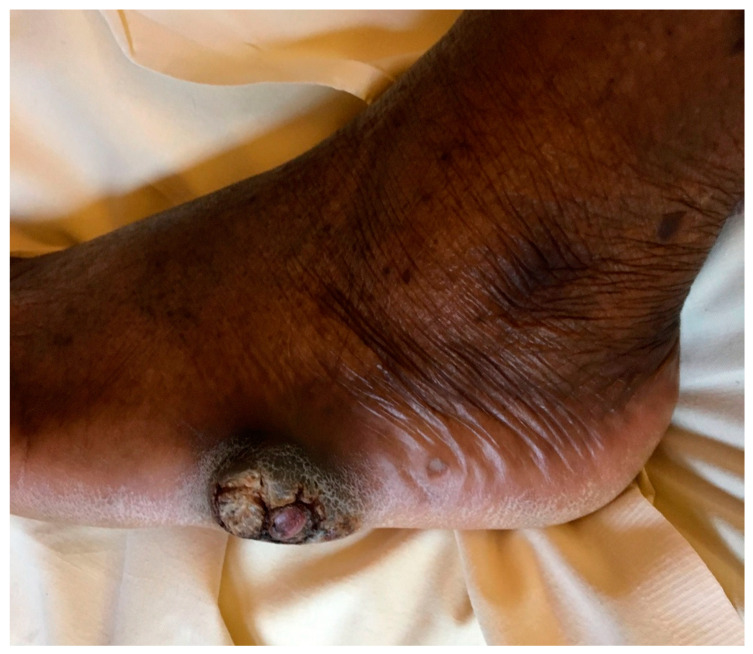
Nodular and warty foot lesion showing pseudoepitheliomatous hyperplasia and hyperkeratosis, after five months of progression.

Three months later (5 months after lesion onset), the lesion grew lumpy and tumoral with pseudoepitheliomatous hyperplasia, reaching 3 cm and becoming increasingly disabling (Figure 1). No evidence of sporotrichoid inflammation or systemic dissemination was found. An X-ray and magnetic resonance imaging of the foot revealed a dermo-hypodermic polylobed mass syndrome with no osteitis lesions. The patient remained in good general condition, with no fever or biological inflammatory syndrome.

**Figure 2:**
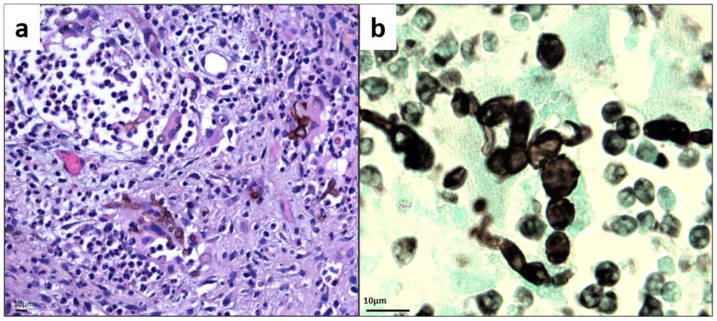
Histology of skin biopsy (**a**) Hematoxylin Eosin Safran stain (magnification ×500). (**b**) Gomori–Grocott stain showing bloated hyphae and chain-organized muriform cells characterized by a round to polyhedral (chestnut) shape, 5 to 12 mm in diameter, with a thick wall, a dark pigment, and both transverse and longitudinal cross-walls (magnification ×1000).

Histologically, the skin biopsy revealed a deep abscessed lesion composed of many altered polynuclear cells, histiocytes, and a few giant cells. Numerous dark pigmented fungal spores and inconsistently branched swollen filaments of varying size, evocative of phaeohyphomycosis, were observed, Figure 2a. Concomitantly, mycological examination of potassium hydroxide mounts revealed the presence of many dematiaceous muriform cells. Histopathological examination confirmed the presence of such pathognomonic muriform elements within the tissue, sometimes organized as chains, thus clarifying the diagnosis of chromoblastomycosis, Figure 2b.

**Figure 3:**
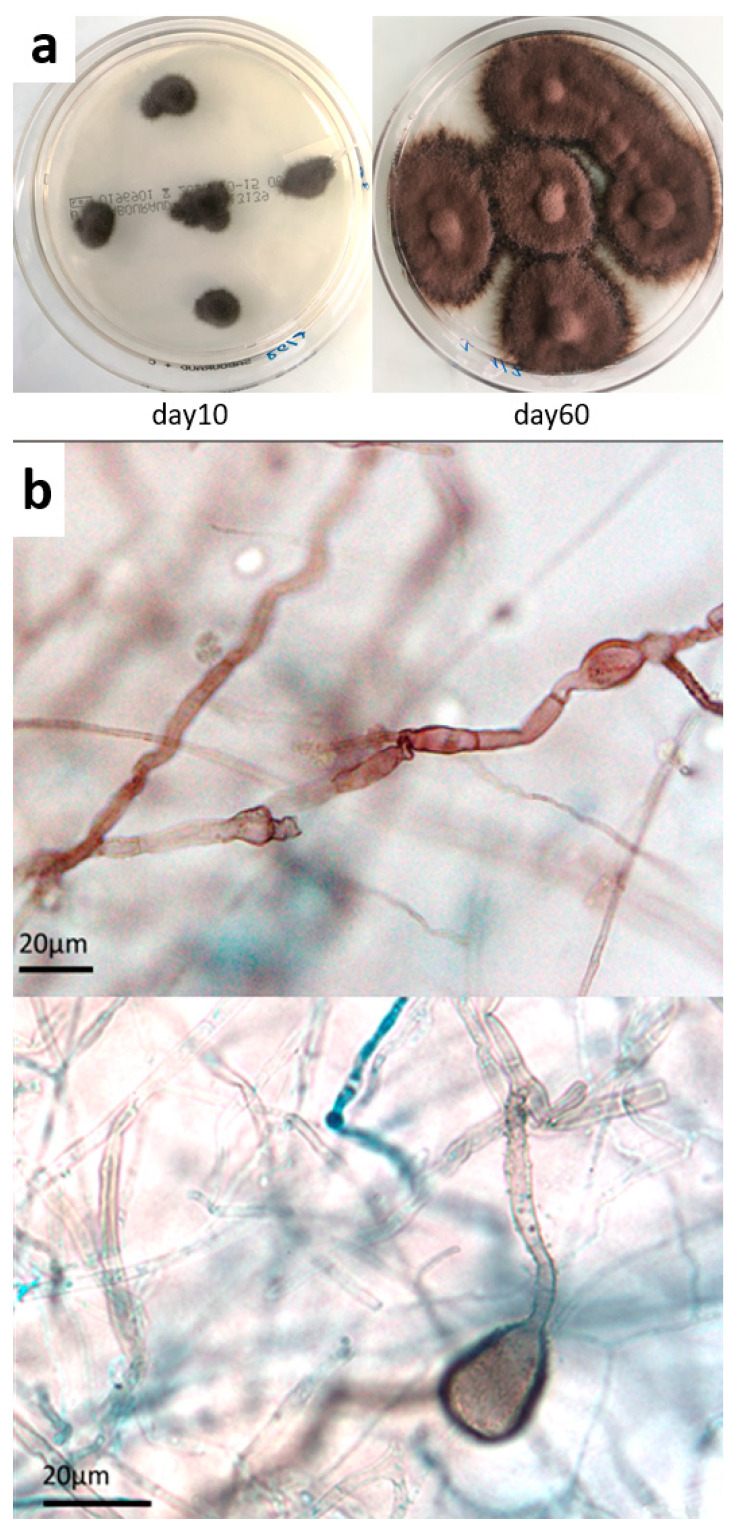
Mycological culture of skin biopsy. (**a**) Colonies obtained on Sabouraud chloramphenicol plates after incubation at 27 °C for 10 and 60 days. (**b**) Microscopic appearance of fungal colonies, showing dark sterile hyphae with swellings and cell-wall ornamentations (magnification ×500, ×1000, respectively).

Mycological cultures from both skin biopsies grew in 10 days, with numerous dark pigmented colonies, Figure 3a, yielding poor microscopic features. Smooth black hyphae with no remarkable fructifications were observed, apart from large echinulate chlamydospores and balloon-like filaments appearing in older cultures Figure 3b.

**Figure 4:**
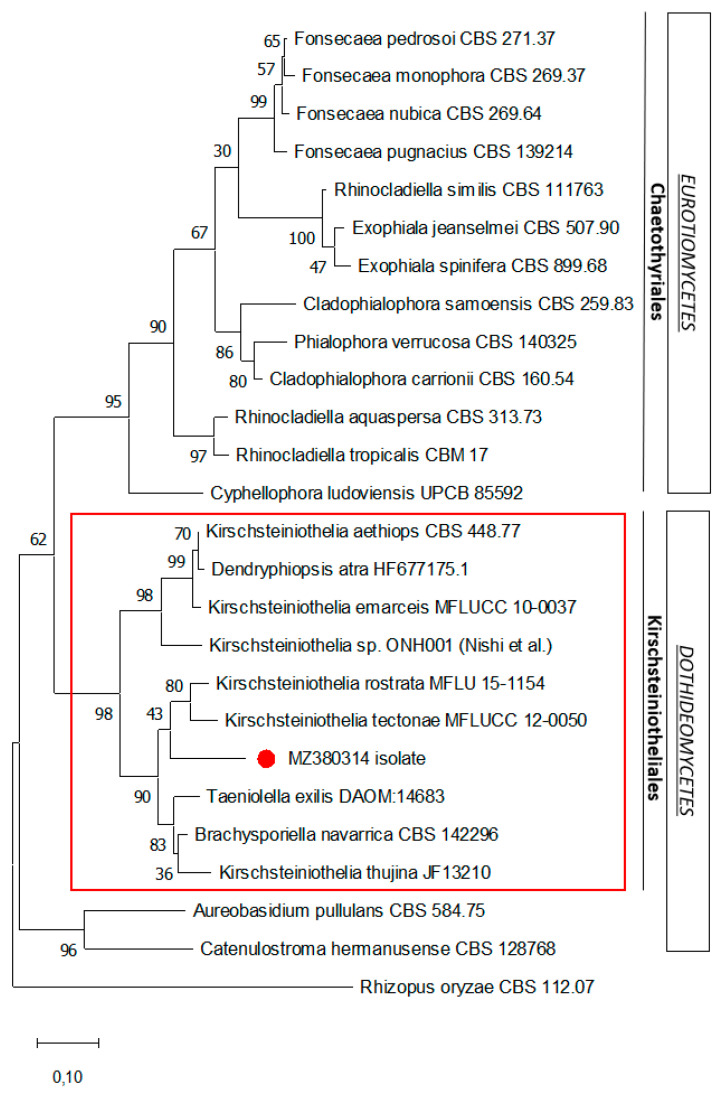
Maximum likelihood phylogenetic analysis of the ITS1-5.8S-ITS2 rDNA sequence of the isolate. The phylogenetic tree was constructed using the MEGA X program. The sequence related to this case is indicated by the red dot. Numerical values on the branches are the bootstrap values given as the percentage of bootstrap replications from a 2000 replicate analysis. The scale bar represents the number of substitutions per site. *Rhizopus oryzae* is used as an outgroup.

Molecular sequencing of the ITS1-5.8S-ITS2 and 28S (LSU) regions of the ribosomal DNA was performed and the nucleotide sequences deposited in Genbank (ITS: MZ380314 and LSU: MZ380317). Comparison with fungal species from the Genbank database showed a high percentage of identity matches with the Dothideomycetes class, especially with Kirschsteiniotheliales, such as *Kirschsteiniothelia* spp. (88–95%), *Taeniolella exilis* (94.6%), and *Dendryphiopsis* spp. (89.13–89.48%). A phylogenetic analysis of the ITS1-5.8S-ITS2 rDNA sequence was performed (Figure 4), showing that the sequenced isolate was a member of Kirschsteiniotheliales (Dothideomycetes) and unrelated to the Chaetotyriales taxon (Eurotiomycetes), of which most species commonly responsible for chromoblastomycosis belong. However, no reliable species identification could be attributed, probably because this fungus has never been described.

As there was no local spreading, nor to the bone, excision surgery of the entire lesion was performed, and the patient received post-operative antifungal therapy with liposomal amphotericin B (3 mg/kg) for 10 days. Clinical follow-up at 6, 12, and 24 months after treatment showed complete healing of the lesion, despite the persistence of residual difficulty in walking for a few months due to a hyperkeratosis reaction at the resection scar.

## 3. Discussion

Several possible causes of abscess-like skin lesion were considered for this immunocompromised patient, such as carcinoma, Kaposi disease, or an infectious abscess, but the fungal origin of the disease was rapidly confirmed. However, the accurate mycological diagnosis was complicated due to (i) the absence of typical muriform cells on first examination, (ii) the absence of obvious vegetal foot trauma, and iii) the absence of 100% matching DNA sequences in current mycological databases, which prevented us from precisely identifying the fungal species and, thus, from experience-guided therapeutic management.

From a mycological point of view, this is the first case of histopathology-proven chromoblastomycosis caused by a dematiaceous fungus not belonging to the Chaetotyriales order (Eurotiomycetes)—in particular, the Herpetrichiellaceae family, which includes the well-known genera *Fonsecaea*, *Cladophialophora*, *Rhinocladiella*, and *Exophiala*. Several unexpected agents, such as *Aureobasidium pullulans*, *Rhytidhysteron* spp., *Chaetomium funicola*, and *Catenulostroma chromoblastomycosum*, have been previously shown to be responsible for chromoblastomycosis-like skin lesions, albeit with no evidence of muriform cells in most cases [6]. Interestingly, Nishi et al. recently reported the first human infection due to a phenotypically similar mold, identified as the *Kirschsteiniothelia* species, in a Japanese patient presenting with infectious ankle bursitis [7] with no clinical presentation of chromoblastomycosis. Although phenotypically similar, the isolate analyzed in our study shares less than 90% DNA similarity with their strain, suggesting that it is a species that has never been described.

Recently introduced, the Kirschsteiniotheliales order is comprised of a diverse panel of environmental saprophytic species isolated from decaying wood, herbaceous debris, soil, and water. The few environmental reports of *Kirschsteiniothelia* come from Asia [8] and Europe, especially from the South of France and Spain [9,10,11]. In our case, it is likely that the patient was infected in Morocco by walking barefoot and inoculation with an imperceptible splinter that went unnoticed at the time.

Melanized saprophytic fungi are distributed worldwide, especially in extreme conditions, including in decaying wood or leaves and soil. Although the pathogenicity of such dematiaceous fungi is generally considered to be limited, they probably possess the properties required for virulence towards plant or human tissues and low susceptibility to antifungals [12]. There are very scarce data on the antifungal susceptibility of Kirschsteiniotheliales due to their rare involvement in human infections, as well as technical issues related to their slow growth in vitro. A *Kirschsteiniothelia* spp. isolate was tested by Nishi et al. and showed a high minimal inhibitory concentration (MIC) against most of the usual antifungals, such as triazoles (>8 µg/mL for itraconazole and voriconazole), amphotericin B (>16 µg/mL), and echinocandins (0.5 µg/mL and 4 µg/mL, for micafungin and caspofungin, respectively) [7]. By contrast, the MIC against 5-fluorocytosin, which can be combined with azoles, was low (0.12 µg/mL). In any case, such low overall antifungal susceptibility supports the need to include physical treatment in the management of chromoblastomycosis, e.g., thermotherapy or phototherapy, when early surgical excision of the initial lesion is not performed [13].

Chromoblastomycosis still presents a true therapeutic challenge due to the recalcitrant nature of the disease, prone to frequent recurrence. The present case was successfully treated as a result of surgery and early medical management and the absence of bone involvement, but the evolution of the disease in this transplant patient could have been dramatic.

In conclusion, this original case illustrates the potential diversity of environmental dematiaceous fungi responsible for phaeohyphomycosis, especially chromoblastomycosis, and the need to send samples to mycology lab for appropriate diagnosis.

## Data Availability

All the available data were reported in the case presentation.
